# Correlation analysis between quality characteristics and rhizosphere microorganisms of different wine grape varieties during their ripening phase

**DOI:** 10.3389/fmicb.2025.1546323

**Published:** 2025-03-26

**Authors:** Dao-Jun Guo, Dong-Ping Li, Meng-Yu Zhang, Yong-Le Wu, Guo-Rong Yang, Zhi-Fang Liu, Yang Jiao, Bin Yang

**Affiliations:** ^1^College of Life Sciences and Engineering, Hexi University, Zhangye, China; ^2^Biodiversity Science Popularization Base of Hexi Corridor, Hexi University, Zhangye, China; ^3^Institute of Hexi Ecology, Hexi University, Zhangye, China; ^4^College of Life Sciences, Sichuan Agricultural University, Ya’an, China

**Keywords:** wine grapes, organic acid, phenols, soil microorganisms, endogenous microorganisms

## Abstract

Wine grapes are the raw material used in wine brewing. The soil microenvironment is regulated by plant rhizosphere microorganisms, which can have a direct or indirect impact on plant growth and development. The population distribution of rhizosphere soil and endophytic microorganisms of Cabernet Sauvignon, Merlot, and Pinot Noir was investigated in this study utilizing high-throughput sequencing technology in relation to the characteristics of wine quality during the ripening phase. The results showed that the community composition of dominant fungi and bacteria in the rhizospheric soil of the three wine grapes varieties was similar at the phylum level. The microbial richness of Cabernet Sauvignon rhizosphere soil was higher than that of Merlot and Pinot Noir, and the bacterial community structure of various wine grape rhizosphere soil varied at the genus level. There were more differential microorganisms in rhizosphere soil than endophytic microorganisms. At the phylum level, malic acid correlated favorably with Mortierellomycota, while flavonol in the fruit peel and flesh of wine grapes correlated favorably with Aphelidiomyceta and Calcarisporiellomycota in rhizosphere soil fungi; The fruit peel’s malic acid showed a negative correlation with the soil bacterial community’s verrucomicrobiota, while the fruit flesh’s succinic and oxalate acids showed a favorable correlation. Proanthocyanidin in wine grape fruit flesh positively correlated with several fungal genera in rhizosphere soil at the genus level, including *Hydnocystis*, *Schizothecium*. Additionally, there was more negative correlation than positive correlation between wine grape quality and soil bacterial community. Several endophytic fungal communities showed good correlations with the proanthocyanidin in wine grapefruit flesh. The fruit peel’s ascorbic acid, phenolics, and tannins showed a favorable correlation with rhizosphere endophytic bacteria that were highly abundant at the genus level. However, some endophytic bacteria negatively correlated with malic acid in the fruit flesh. This study provides new ideas and theoretical support for improving the quality of grapes for winemaking.

## 1 Introduction

Merlot, Cabernet Sauvignon, and Pinot Noir are three common grape varieties used in winemaking, widely grown in the Hexi Corridor region of China (Landbo and Meyer, 2001). The climate in this region is similar to that of world-renowned wine-producing areas such as Bordeaux in France (Yan et al., 2022; Jakabová et al., 2021). The region has a high annual total solar radiation, with an average annual sunshine duration of over 3,000 h, a significant temperature difference between day and night, moderate annual precipitation, and high evaporation, which is favorable to the accumulation of sugar and flavor formation of grapes, providing unique geographical conditions for the growth of wine grapes (Wang et al., 2018; Zhang et al., 2023; Hu et al., 2021). The rhizosphere of plants is an essential active area of microorganisms, including rhizosphere soil and roots (Katznelson et al., 1948; Liu et al., 2023; Dotaniya and Meena, 2015). Rhizosphere microorganisms, as a critical component of soil ecosystems, are closely related to the growth and quality of crops (Avis et al., 2008; Babalola et al., 2021). It promotes crop absorption and utilization of nutrients through biological nitrogen fixation, secretion of organic acids, enzymes, antimicrobial peptides, and plant hormones, and dissolution of insoluble minerals in the soil, improving crops’ nutritional value and quality (Xiong et al., 2021; Kokalis-Burelle et al., 2006). The grape rhizosphere microbial community comprises various microorganisms, mainly bacteria, fungi, and actinomycetes, as well as a few protozoa and algae (Zhang et al., 2019). Among them, bacteria are the most diverse group and their number in the soil is usually several times or even dozens of times higher than that in non-hizosphere soil (Singh et al., 2022). Fungi and actinomycetes also play a crucial role in soil structure and nutrient transformation in grape rhizosphere soil (Novello et al., 2017; Guo et al., 2011). The composition of the rhizosphere microbial community in wine grapes is not fixed and unchanging but changes with various factors such as grape growth cycle, soil type, climate conditions, and cultivation management measures. For example, the composition and quantity of rhizosphere microbial communities vary during different growth stages of grapes, such as germination, flowering, fruiting, etc. (Bao et al., 2022; Oyuela Aguilar et al., 2020; Zhang et al., 2019; Vega-Avila et al., 2015; Wang et al., 2024). Rhizosphere microorganisms affect the metabolic activity of grape roots, which affects the quality of grapes. For example, certain microorganisms can increase the sugar content and aroma components in grapes, improving the quality of wine production (Wei et al., 2022; Rezende et al., 2024). Various factors, including soil type, climatic conditions, cultivation management measures, and grape varieties, influence the composition and function of grape rhizosphere microorganisms (Wei et al., 2018; Zahid et al., 2022; Compant et al., 2019).

Acid and phenolic substances during the ripening period of wine grapes are essential factors affecting the quality of wine grapes. Acidic substances such as tartaric, malic, and citric acid give the wine its taste and affect its acidity and aging potential (Mato et al., 2005; Liu et al., 2022; Zhai et al., 2023). During the ripening process of grapes, the content of these acidic substances will change. Moderate acidity can balance the sweetness, alcohol content, and other flavor substances of wine, making the taste of wine more sweet (Shiraishi, 1995; Milovanovic et al., 2019; Vicente et al., 2022). Phenolic substances, including anthocyanins, tannins, etc., significantly contribute to wine’s color, taste, and aroma (Waterhouse, 2002). As grapes mature, the content and types of phenolic substances also change, directly affecting wine’s flavor complexity. Tannins not only bring astringency to wine but also react with other components of wine (Tu et al., 2022; Zhao et al., 2023a, 2023b). Therefore, obtaining the appropriate content of acids and phenols is one of the critical factors in producing high-quality wine. Researchers have analyzed the quality of wine grapes from different varieties, and there are significant differences in the quality of wine produced from different wine grapes in the same region (Král et al., 2018; Jakabová et al., 2021).

By breaking down organic materials, rhizosphere microbes can indirectly alter soil acidity, impacting how well acidic chemicals are absorbed and transported by grape roots (Barata et al., 2012; Liu et al., 2019). In addition, it takes part in the cycling and transformation of soil nutrients, which may impact how well grape plants absorb and use minerals, indirectly affecting the production and build-up of phenolic compounds in grapefruits (Gabriele et al., 2016; Berríos et al., 2024). Microbial ecology also significantly impacts wine quality throughout the winemaking process. In addition, microbial ecology also significantly impacts wine quality throughout the winemaking process (Liu et al., 2019; Englezos et al., 2022). Thus, it is crucial to comprehend the relationship between alterations in grape acids phenols and microorganisms in the rhizosphere for grapes used to make wine. However, there is still limited research on whether the rhizosphere soil microbial communities of different wine grapes affect grape quality, whether there are differences in winemaking, and the correlation between microbial community distribution and grape quality. This study used high-throughput sequencing techniques to examine the diversity of rhizosphere soil and root microorganisms in three mature grape varieties—Pinto Noir, Cabernet Sauvignon, and Merlot. The relationship between quality variations in grape varieties and rhizosphere microorganisms was shown by analyzing the variations in acidic and phenolic chemicals across several grape varieties using a combination of high-performance liquid chromatography and biochemical approaches. Our findings provide the significant impact of rhizospheric microorganisms on improving grape wine quality.

## 2 Materials and methods

### 2.1 Sampling of wine grape rhizosphere soil and roots

The rhizospheric soil and roots of Merlot, Cabernet Sauvignon, and Pinot Noir were collected from the planting base of Guofeng grape wine industry Co., Ltd., located in Banqiao Town, Linze County, Zhangye City, Gansu Province, China (100°19′49″ E, 3915′22″N), with an average altitude of about 1,400 m, an annual effective accumulated temperature above 10°C is 3,053°C, the frost-free period is 160 days; It is dry and rainless, with an average annual rainfall of 120 mm, an average annual temperature of 8.1°C, an average daily temperature of 14.9°C, a significant temperature difference between day and night, and a long sunshine duration. The rhizospheric dirt from wine grapes was removed using a sterile brush before being placed in a sterile container. After 2 min of treatment with 75% alcohol, the grape roots were put in sterile bags and kept at −80°C for later use. Triplicate rhizospheric soil and root samples were collected from each of the three grape varieties: Merlot, rhizospheric soil samples (MLS: MLS1, MLS2, MLS3), root samples (MLR: MLR1, MLR1, MLR3) Cabernet Sauvignon, rhizospheric soil samples (CSS: CSS1, CSS2, CSS3), root samples (CSR: CSR1, CSR1, CSR3); Pinot Noir, rhizospheric soil samples (PNS: PNS1, PNS2, PNS3), and root samples (PNR: PNR1, PNR3). Similarly, grape samples were collected from three wine grape varieties at maturity, weighing approximately 3 kilograms, and brought back to the laboratory to manually classify the fruit peel and flesh before storing them at −80°C for testing.

### 2.2 Determination of acids in grape by high-performance liquid chromatography

High-performance liquid chromatography (HPLC) was used to detect the organic acids in the fruit peel and flesh of three wine grape varieties, such as oxalate acid (OAP, OAF), tartaric acid (TAP, TAF), malic acid (MAP, MAF), ascorbic acid (AAP, AAF), citric acid (CAP, CAF), and succinic acid (SAP, SAF). Weigh 0.25 g of powdered grape peel precisely, then dilute it with ultrapure water in a 5 mL centrifuge tube and centrifuge it for 10 min at 10,000 rpm at 4°C. The supernatant was used for standby. 1 mL of supernatant was added to an equal volume of phosphoric acid buffer, centrifuged at 4°C and 10,000 R/min for 10 min, and filtered the supernatant through the 0.45 μm filter membrane. The fruit flesh was crushed by a juicer and centrifuged at 4°C for 15 min at 10,000 R/min. 2 mL of the supernatant was added to the mobile phase (1: 99 methanol+0.01 mol/L K_2_HPO_4_ solution) to a constant volume of 10 mL. The mixture was evenly mixed and centrifuged repeatedly once, and the supernatant was filtered with a 0.45 μm filter membrane. The processed samples were analyzed by high-performance liquid chromatography (Thermo Fisher U 3,000). The chromatographic conditions were as follows: the mobile phase a was 0.01 mol/L K_2_HPO_4_ solution (pH 2.4), and the mobile phase B was chromatographic methanol, filtered by 0.45 μm membrane before use, and degassed by ultrasound for 30 min; The flow rate was 0.5 mL/min, the column temperature was 30°C, the injection volume was 10 μLand, and the detection wavelength was 210 nm. The gradient of the organic acid standard solution is shown in Supplementary Table S1, and the chromatogram of the organic acid mixed standard solution is listed in Supplementary Figure S1.

### 2.3 Detection of phenolic substances in wine grapes

Some phenolic substances of tannin (TP, TF), flavonol (FlP, FlF), proanthecyanin (PrP, PrF), and total phenolic (PhP, PhF) in the wine grapes peel and flesh were analyzed based on previous research by Kaufman et al. (2013). After accurately weighing the grape fruit sample to 0.25 g, it was transferred to a 10 mL centrifuge tube, filled with 7.5 mL of 60% acetone solution, and placed in a water bath at 70°C for 7 h. After centrifuging it for 10 min at 8000 r/min, the supernatant was poured into a 30 mL centrifuge tube. To finish the previous steps, thoroughly mixed the two extracted supernatants, transferred the combined supernatant into a 50 mL volumetric flask, and replaced the lost volume with deionized water.

The assays of total phenolic content in wine grape peel and flesh were performed as previously explained in the work of Hellin et al. (2010). Accurately weighed 0.5 g of grape sample in 7 mL of 80% methanol solution, sonicated at 40°C and 40 kHz for 40 min, and centrifuged at 4000 r/min for 20 min to separate the upper clear liquid. Extracted twice under the same conditions, combined the extracts, transferred the mixed extracts to a 50 mL volumetric flask, and diluted with distilled water.

The evaluation of flavonol content in wine grapes was based on previous reports (Kennedy et al., 2002). The proanthocyanidins were obtained using an iron salt-catalyzed colorimetric method (Pekić et al., 1998). Accurately weighed 0.25 g of grape, added 2.5 mL of extraction solution (acetone: water: formic acid = 80:19; 1), shook in a water bath at 30°C for 30 min, centrifuged to obtain the supernatant, repeat three times, combining the supernatant extracted three times, and mixed thoroughly and evenly.

### 2.4 Extractoin and sequencing of microbial DNA from rhizospheric soil and root samples

DNA was extracted from the rhizospheric soil and root samples of three wine grape varieties using DNA extraction kit (Omega Bio Tec, Norcross, GA, U.S.). The DNA quality was evaluated by 1% agarose gel electrophoresis method. High-throughput sequencing project for MiSeq was completed by Shanghai Meiji Biomedical Technology Co., Ltd. in China. Sequences of primers used for fungal amplification were F: 5 'ACTCCTACGG GAGGCAGCAG-3' and R: 5 'GGACTACHVGG GT WT CTAAT-3' (Martin and Rygiewicz, 2005). The hypervariable region V3-V4 of the bacterial 16S rRNA gene were amplified with primer pairs 5'-CTTGTCATTTAGAAGTA-3', and R: 5'-GCTGCG TTCTTCA TCGATG C-3' in this study (Tringe and Hugenholtz, 2008). The depth of DNA sequencing is shown in Supplementary Table S2. The raw metagenomic sequencing data were submitted to the NCBI SRA database and with the accession number PRJNA1197982 (http://www.ncbi.nlm.nih.gov/ bioproject/1197982).

### 2.5 Library construction and bioinformatics analysis

The raw data obtained by sequencing was spliced and filtered to obtain adequate data (clean data). Dada2 (divisive amplitude denoising algorithm 2) was applied to the legitimate data to minimize noise and filter out sequences with an abundance of less than 5 (Harbuzov et al., 2022). The representative sequence for OTUs (operational taxonomic units) was represented by the final ASVS (amplicon sequence variants) (Supplementary Table S2). One way to retrieve the related species information and abundance distribution for the produced ASVS is to annotate each ASV’s representative sequence. Conversely, the Meiji bio cloud platform in Shanghai, China^1^ was utilized to examine the Venn diagram, alpha diversity, beta diversity, and abundance of ASVS. Principal coordinates analysis (PCoA), dimension reduction analysis, and sample clustering tree display were used simultaneously to investigate the variations in community structure between samples (Liu et al., 2020). *T*-tests, metastat, lefse, and other statistical analysis techniques were used to assess further the significance of variations in species composition and community structure of grouped samples and investigate the differences in community structure among grouped samples.

### 2.6 Data analysis

Excel 2010 was used to summarize the data. SPSS 21 was used to do a one-way ANOVA of the grape quality and correlation analysis between the microbial community and wine grapes’ acid and phenolic content. All figures were plotted using AI 2020 and Origin 2021, and significant differences (*p* < 0.05) were noted.

## 3 Results

### 3.1 Acid and phenolic compounds content analysis in wine grape at ripening stage

The HPLC was used to test the acids in three wine grape varieties at the mature stage. The results showed that no significant difference was observed in oxalic acid and citric acid contents of the grape peel among the three wine grape varieties. Whereas, a significant difference was observed in tartaric acid, malic acid, and succinic acid content. All Cabernet Sauvignon and Pinot Noir varietals showed no discernible differences, although there were some noticeable differences compared to Merlot (Table 1). In addition, there was no significant difference in the malic acid content in the flesh of the three wine grape varieties. However, there were significant differences in the other five acids’ content, and the ascorbic acid change was more considerable in the three wine grape varieties (Table 1). Therefore, there are differences in acid substances among wine grape varieties during the maturity stage.

**Table 1:** Organic acid content in the peel and flesh of wine grapes at maturity.

Sample	Fruit tissue	Oxalate acid (g/L)	Tartaric acid (g/L)	Malic acid (g/L)	Ascorbic acid (g/L)	Citric acid (g/L)	Succinic acid (g/L)
ML	Fruit Peel	0.008 ± 0.004^a^	0.519 ± 0.073^b^	0.072 ± 0.018^c^	0.082 ± 0.032^b^	0.045 ± 0.011^a^	0.286 ± 0.062^b^
CS		0.009 ± 0.005^a^	1.008 ± 0.126^a^	0.365 ± 0.016^a^	0.248 ± 0.254^a^	0.049 ± 0.008^a^	0.473 ± 0.046^a^
PN		0.011 ± 0.001^a^	0.950 ± 0.113^a^	0.231 ± 0.097^b^	0.258 ± 0.149^a^	0.059 ± 0.009^a^	0.323 ± 0.278^a^
ML	Fruit Flesh	0.094 ± 0.049^a^	0.735 ± 0.030^a^	0.831 ± 0.011^a^	0.395 ± 0.069^a^	0.103 ± 0.003^a^	0.178 ± 0.075a
CS		0.008 ± 0.003^b^	0.644 ± 0.020^b^	0.945 ± 0.133^a^	0.084 ± 0.015^b^	0.071 ± 0.002^b^	0.010 ± 0.007^b^
PN		0.041 ± 0.013^ab^	0.718 ± 0.054^ab^	0.887 ± 0.333^a^	0.256 ± 0.089^a^	0.104 ± 0.001^a^	0.027 ± 0.017^b^

The display of lowercase letters in the same column varied significantly (*p* < 0.05), and all data points were mean ± SE (*n* = 3).

**Figure 1:**
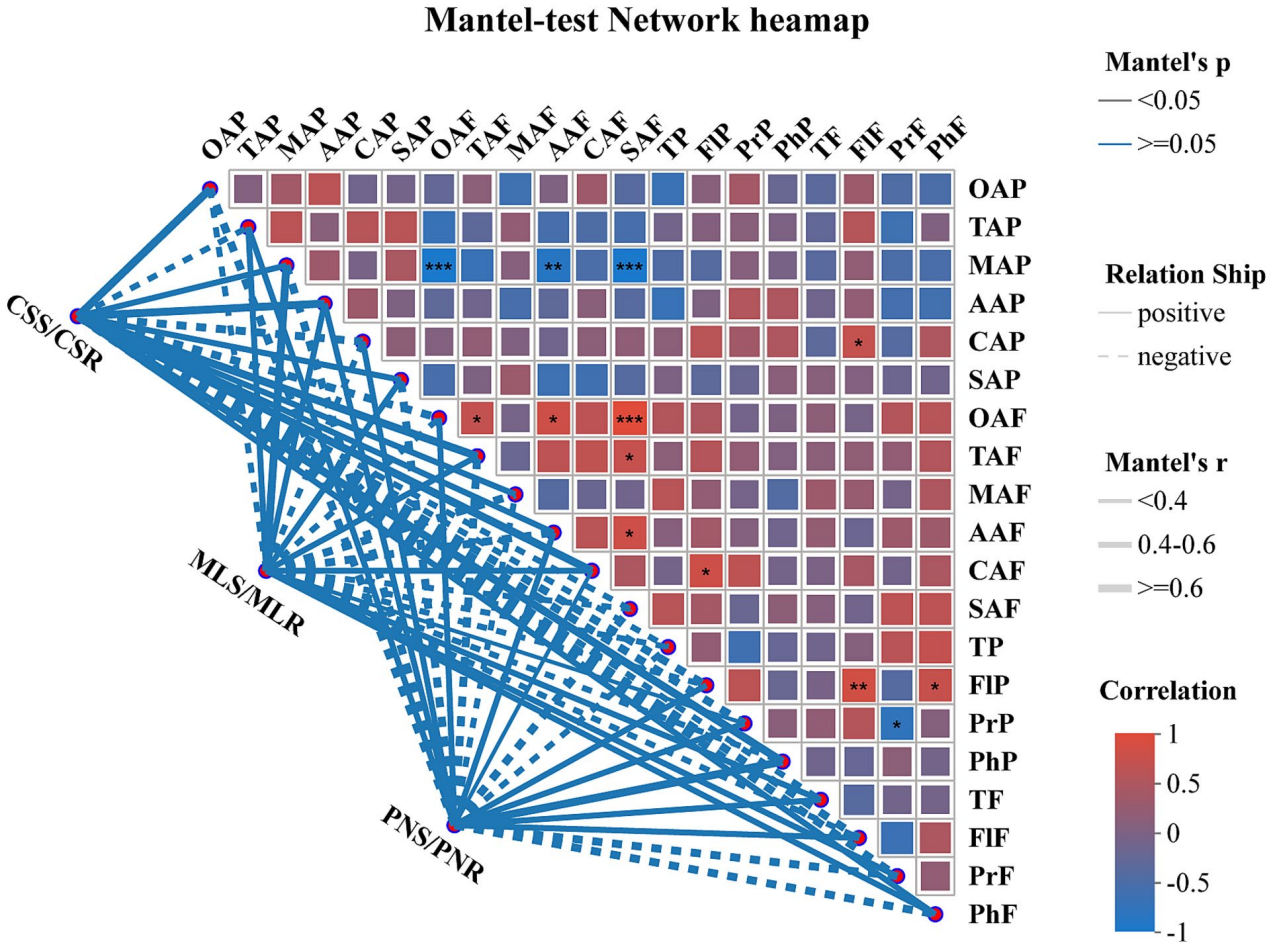
Mantel-test network heatmap analysis of organic acids and phenolic substances in wine grapes and samples. The thickness of the lines in the figure represents the correlation between the sample and the quality indicators of wine grapes. The depth of colors in the heatmap symbolizes the magnitude of positive and negative correlation, while the stars in the color blocks represent significance (*: 0.01 < *p* ≤ 0.05, **: 0.001 < *p* ≤ 0.01, ***: *p* ≤ 0.001).

The detection of phenolic content in the fruit peel of three wine grape varieties at the maturity stage showed no significant difference in tannin and total phenol content among the samples. Nonetheless, the samples’ levels of Proanthocyanidin and flavonol varied considerably, with Pinot Noir having the most significant levels of both phenols (Table 2). Additionally, in the fruit flesh, only tannin had no significant change in the three wine grape varieties. At the same time, flavonol, proanthocyanidins, and total phenols showed significant differences, and the proanthocyanidin concentration varied considerably across all samples (Table 2). The Mantel-test network text analysis showed that oxalate acid, ascorbic acid, and succinic acid in wine grape peel were negatively correlated with malic acid in fruit peel (*p* < 0.001), while succinic acid and oxalate acid in fruit flesh were negatively correlated (*p* < 0.01) (Figure 1).

**Table 2:** Phenolic content in the peel and flesh of wine grapes at maturity.

Sample	Fruit tissue	Tannin (mg/g)	Flavonol (mg/g)	Proanthocyanidin (mg/g)	Total phenolic (mg/g)
ML	Fruit Peel	5.651 ± 0.330^a^	0.370 ± 0.018^b^	1.600 ± 0.009^b^	20.900 ± 3.352^a^
CS		5.350 ± 1.098^a^	0.341 ± 0.018^a^	1.600 ± 0.018^b^	20.600 ± 1.447^a^
PN		4.953 ± 0.739^a^	0.431 ± 0.018^a^	1.770 ± 0.019^a^	20.240 ± 3.345^a^
ML	Fruit Flesh	0.462 ± 0.080 ^a^	0.016 ± 0.001^b^	0.031 ± 0.002^a^	9.050 ± 2.458^a^
CS		0.383 ± 0.149 ^a^	0.017 ± 0.002^b^	0.007 ± 0.000^b^	5.150 ± 2.959^b^
PN		0.453 ± 0.030 ^a^	0.025 ± 0.003^a^	0.001 ± 0.000^c^	9.240 ± 3.1.03^a^

The display of lowercase letters in the same column varied significantly (*p* < 0.05), and all data points were mean ± SE (*n* = 3).

### 3.2 Sequencing results of rhizosphere microbial library of different wine grape varieties

Using IIIumina Miseq high-throughput sequencing, 36 samples of rhizosphere soil and root samples of three different wine grape varieties were sequenced. The information was optimized and filtered. It was shown that the effective sequences of the roots and rhizosphere soil of various wine grape varietals varied (Supplementary Table S2). These included a range of 110,833 to 179,925 for the number of adequate rhizospheric soil fungal sequences, 53 to 538 for sequence length, and 185 to 244 for OTU counts. Rhizospheric soil bacteria had between 55,558 and 85,027 effective sequences, with sequences ranging from 218 to 534 and 2,980 to 3,329 OTU. The effective endophytic fungal sequence of wine grape roots ranged from 77,282 to 100,697, sequence lengths ranged from 50 to 500, and OTU numbers ranged from 30 to 591. The range of endophytic bacterial effective sequences in grape roots was 88,060–111,900, the range of sequence lengths was 325–466, and the range of OTU was 3,290–3,486 (Supplementary Table S2). The sequencing results genuinely reflect the microbial community in the sample.

### 3.3 Diversity analysis of rhizosphere microbial populations of different wine grape varieties

The diversity of effective sequences was analyzed at a 97% similarity level. The taxonomic numbers of fungi and bacteria in the samples at phylum, class, order, family, and genus levels were counted (Supplementary Table S3). The Shannon and Simpson indices of fungal communities in the rhizosphere soil of three wine grape varieties showed significant differences, with the highest Shannon and lowest Simpson index in the Cabernet Sauvignon variety, indicating a higher fungal richness in the rhizosphere soil of Cabernet Sauvignon compared to the other two varieties (Table 3). The alpha diversity of endophytic bacteria and fungi in wine grape roots varies significantly. It suggests that the microbial community in the rhizospheric soil is less diversified than the endophytic bacteria found in the roots of wine grapes. The Merlot varietal has greater values for the Ace, Chao, and Shannon markers than the other two kinds, while the Simpson value is the lowest (Table 3). This suggests that Merlot has a more varied endophytic fungal and bacterial community than the other two wine grape varieties (Table 3).

**Table 3:** Alpha diversity of rhizosphere microorganisms in wine grapes.

Sample		Fungi				Bacteria			
		Ace	Chao	Shannon	Simpson	Ace	Chao	Shannon	Simpson
ML	Soil	5.667 ± 3.615^a^	8.000 ± 1.459^a^	0.562 ± 0.055^b^	0.752 ± 0.038^a^	3742.314 ± 1986.483^a^	3703.457 ± 162.871^a^	6.631 ± 0.043^a^	0.004 ± 0.001^a^
CS		9.000 ± 0.894^a^	9.000 ± 0.894^a^	0.819 ± 0.105^a^	0.573 ± 0.096^b^	3632.682 ± 196.837^a^	3571.721 ± 158.927^a^	6.000 ± 0.019^a^	0.004 ± 0.000^a^
PN		0.117 ± 0.068^a^	9.333 ± 0.516^a^	0.700 ± 0.131^ab^	0.715 ± 0.065^ab^	3711.420 ± 216.825^a^	3686.873 ± 214.475^a^	6.643 ± 0.101^a^	0.005 ± 0.002^a^
ML	Root	584.945 ± 84.495^a^	568.799 ± 71.839^a^	2.471 ± 0.027^a^	0.145 ± 0.005^b^	584.948 ± 84.495^a^	568.799 ± 71.839^a^	2.471 ± 0.027^a^	0.145 ± 0.005^b^
CS		71.658 ± 32.960^b^	81.174 ± 18.687^b^	1.920 ± 0.487^a^	0.267 ± 0.052^b^	74.324 ± 36.054^b^	74.175 ± 36.597^b^	1.920 ± 0.487^b^	0.267 ± 0.052^b^
PN		63.034 ± 19.881^b^	62.250 ± 20.502^b^	0.870 ± 0.457^b^	0.706 ± 0.147^a^	63.034 ± 19.881^b^	62.250 ± 20.502^a^	0.870 ± 0.457^b^	0.706 ± 0.147^a^

The display of lowercase letters in the same column varied significantly (*p* < 0.05), and all data points were mean ± SE (*n* = 3).

### 3.4 Diversity and richness of rhizosphere microbial communities in different wine grapes

**Figure 2:**
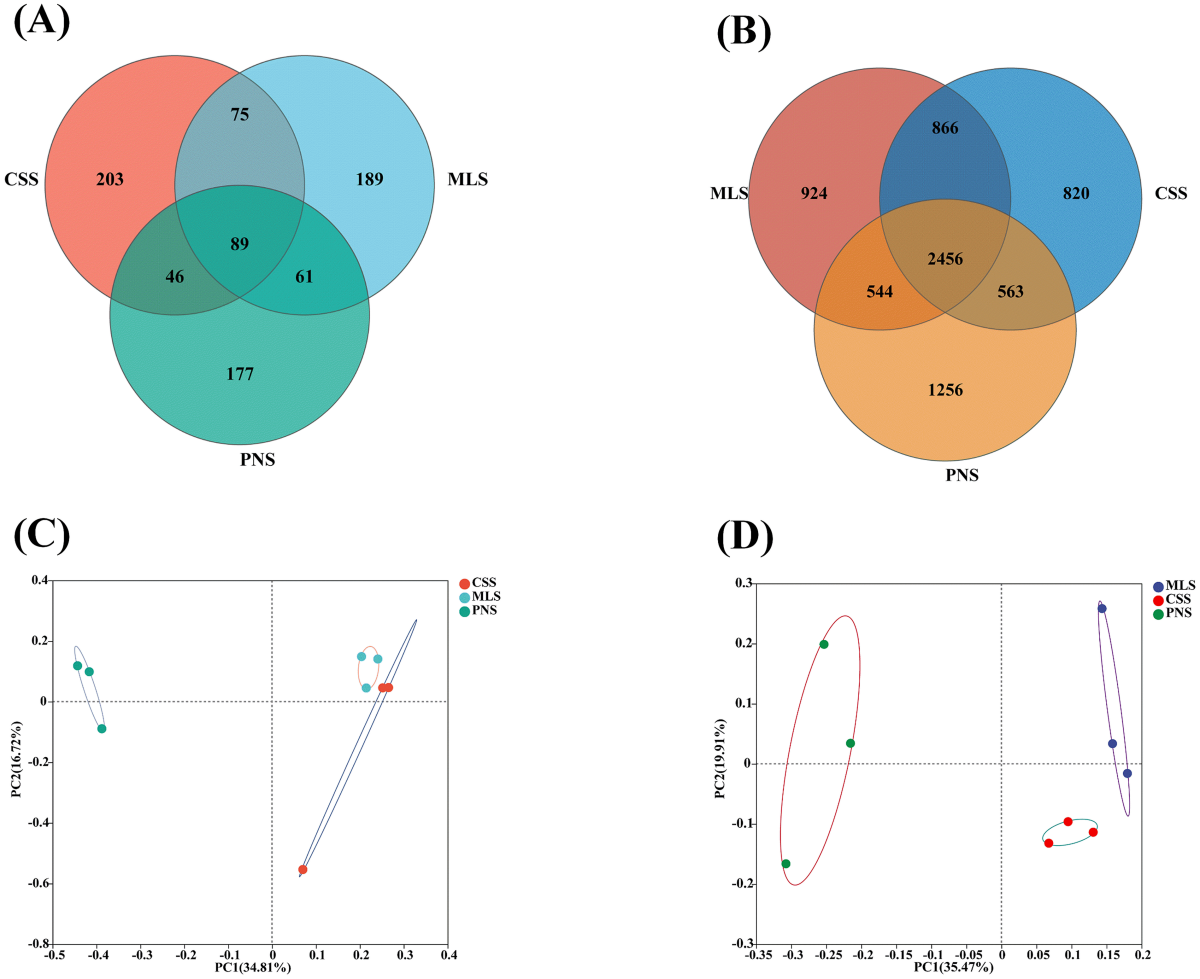
The differences in microbial community structure in the rhizosphere soil of wine grapes. **(A)** Venn of the fungal community. **(B)** Venn of the bacterial community. **(C)** PCoA of the fungal community. **(D)** PCoA of bacterial community.

The OTU quantities of fungi and bacteria in the rhizosphere soil and roots of three wine grapes were analyzed. In the rhizospheric soil fungal community, OTU in PNS was 373, 9.90, and 9.68% less than MLS and CCS, respectively (Figure 2A). However, in the rhizospheric soil bacterial community, the number of OTU in PNS increased by 0.61 and 2.42% compared to MLS and CSS (Figure 2B), indicating that the microbial diversity in the rhizosphere soil of Pinot Noir is greater than that of Merlot and Cabernet Sauvignon. The first and second principal components of rhizospheric soil fungal PCoA analysis explained 34.81 and 16.72% of community variation, respectively (Figure 2C). However, the rhizospheric soil bacterial PCoA analysis explained 35.47 and 19.91% of community variation (Figure 2D). There is a significant separation in the rhizosphere soil samples of three wine grapes, showing notable variations in the microbial composition of the three wine grapes. At the same time, the distance between the Merlot and Cabernet Sauvignon samples is closer than that of the Pinot Noir sample, demonstrating that the microbial composition of the rhizosphere soil of wine grape Cabernet Sauvignon and Merlot is relatively similar to that of Pinot Noir.

**Figure 3:**
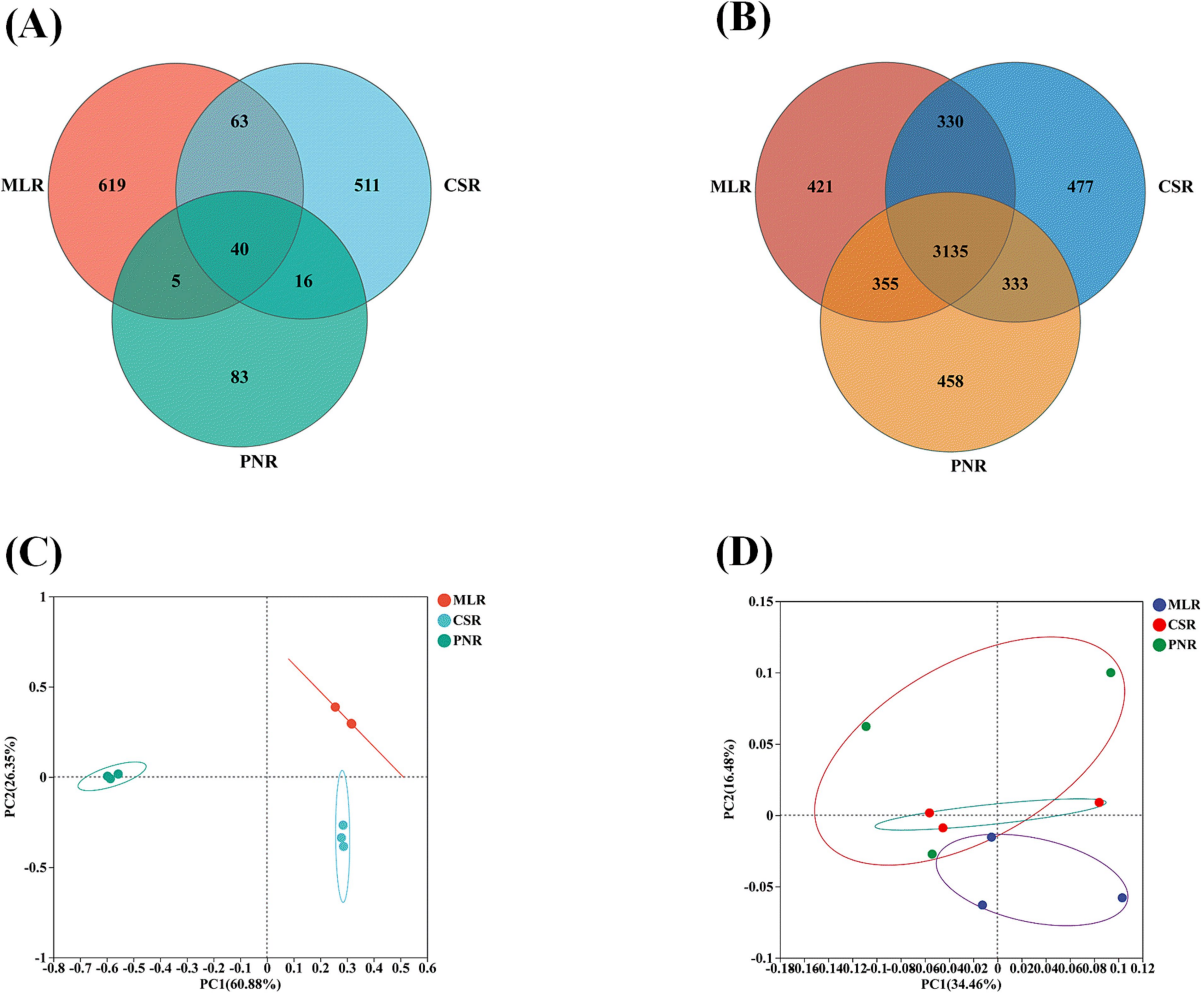
The differences in endophytic community structure of wine grape roots. **(A)** Venn of the endophytic fungal community. **(B)** Venn of the endophytic bacterial community. **(C)** PCoA of the endophytic fungal community. **(D)** PCoA of the endophytic bacterial community.

The endophytic fungal community in wine grape roots, with OTU numbers of 727, 630, and 144 in MLR, CSR, and PNR, respectively, shows significant differences in fungal diversity among the three wine grape roots, particularly in Pinot Noir, where fungal diversity is notably lower than in Cabernet Sauvignon and Merlot (Figure 3A). The difference lies in the endophytic bacterial community, with OUT numbers of 42,434,275 and 4,287 in MLR, CSR, and PNR, indicating that the diversity of endophytic bacteria in wine grape roots is similar (Figure 3B). In addition, the first and second principal components of PCoA analysis of endophytic fungi explained 60.88 and 26.35% of the community variation (Figure 3C). However, endophytic bacteria PcoA community variation explained 34.46 and 16.48% of the community variation (Figure 3D), demonstrating that endophytic fungi have more significant community composition variation than bacteria.

### 3.5 Diversity analysis of dominant microbial communities in wine grapes rhizosphere based on different classification levels

**Figure 4:**
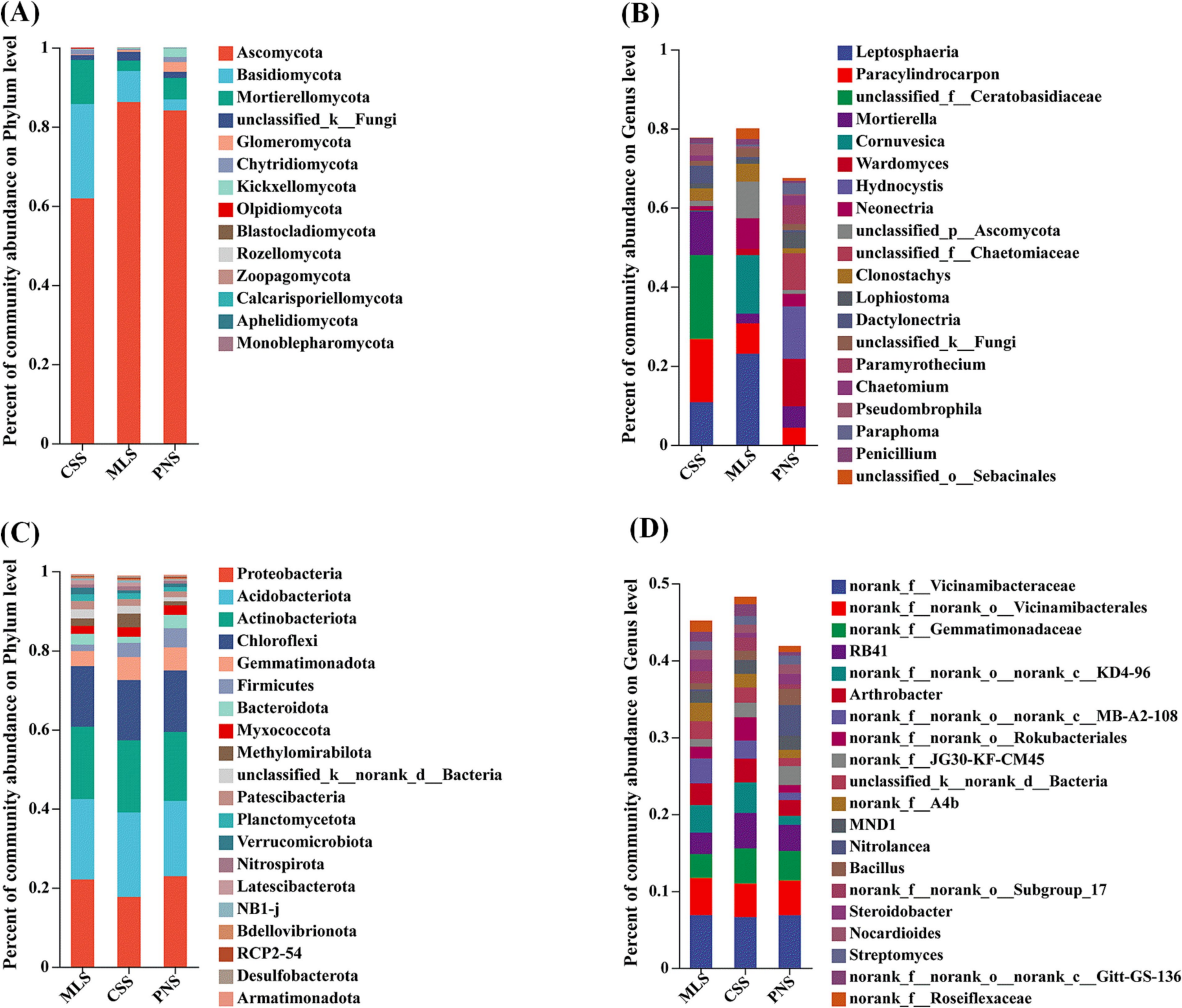
Microbial community composition in the rhizosphere soil of wine grapes. **(A)** Fungal community at the Phylum level. **(B)** Fungal community at the Genus level. **(C)** Bacterial community at the Phylum level. **(D)** Bacterial community at the Genus level.

At the phylum level, the analysis of the TOP20 composition of fungal communities in the rhizosphere soil of different wine grapes showed that the three dominant fungal phyla in the rhizosphere soil of wine grapes were similar, with Ascomycota, Basidiomycota, and Mortierellomycota ranking in the top three categories of microorganisms. The total relative abundance in MLS, CSS, and PNS ranged from 61.90 to 86.24%, 2.78 to 23.83%, and 2.61 to 11.14%, respectively. Among them, Ascomycota has the highest proportion in Merlot samples, while Basidiomycota and Mortierellomycota have the highest proportion in Cabernet Sauvignon (Figure 4A). At the genus level, the three dominant fungal genera in the rhizosphere soil of wine grapes have differences, with important species including *Leptosphaeri*a, *Paracylindrocarpon*, *Mortierella*, *Cornuvesica*, *Wardomyces*, etc. Among them, *Leptosphaeria* has the highest proportion in Merlot, and the abundance of fungal microorganisms in the rhizosphere soil of Merlot is higher than that of Cabernet Sauvignon and Pinot Noir (Figure 4B).

At the phylum level, the analysis of the TOP20 composition of bacterial communities in the rhizosphere soil of different wine grapes showed that the top three categories of microorganisms were Proteobacteria, Acidobacteriota, and Actinobacterota, with relative abundances ranging from 17.69 to 22.93%, 19.02 to 21.33%, and 14.45 to 18.26% in MLS, CSS, and PNS, respectively. The community structures of these three wine grape rhizosphere soil bacterial phyla were similar at the phylum level (Figure 4C). At the genus level, there are differences in the bacterial community structure in the rhizosphere soil of three wine grape varieties, and the microbial richness in the rhizosphere soil of Cabernet Sauvignon is higher than that of Merlot and Pinot Noir (Figure 4D).

**Figure 5:**
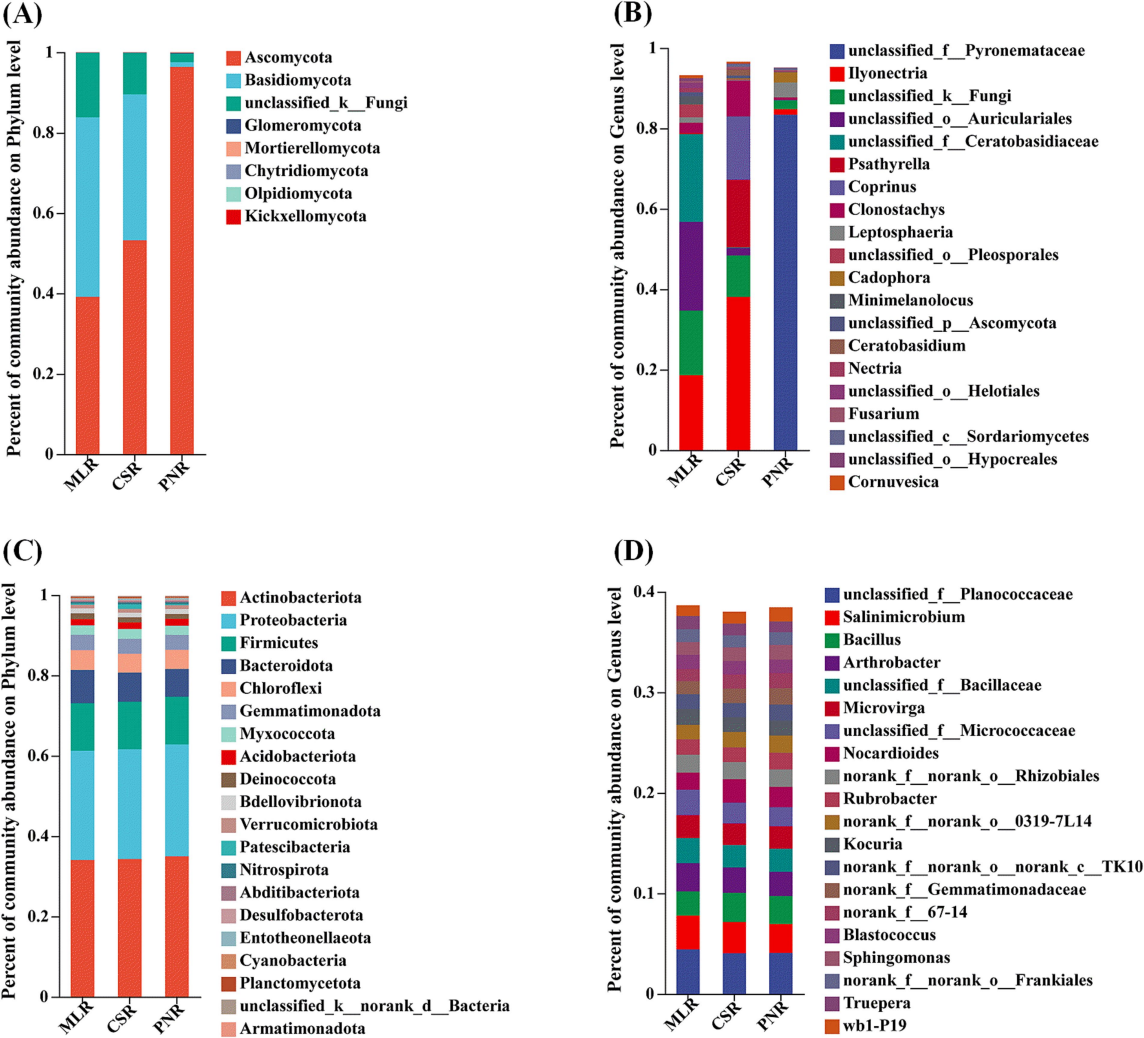
Community composition of endogenous microorganisms in wine grape roots. **(A)** Endophytic Endophytic fungal community at the Phylum level. **(B)** Endophytic fungal community at the Genus level. **(C)** Endophytic bacterial community at the Phylum level. **(D)** Endophytic bacterial community at the Genus level.

The study of the structure of the endophytic fungal community in wine grape roots revealed that the diversity of the endophytic fungal community was low at the phylum level. Ascomycota and Basidiomycota comprised the majority of the community, with Ascomycota accounting for 96.36% of the samples from Pinot Noir (Figure 5A). At the genus level, the endophytic bacterial community structure in the roots of the top 20 samples showed diversity as a whole. The unclassified_f_pyronemataceae without annotation at the genus level accounted for the highest proportion, up to 83.39% (Figure 5B). In addition, the endophytic bacteria in the roots of the three wine grapes have similar community results, and the dominant microorganisms with a high proportion are actinobacteria, Proteobacteria, Firmicutes, bacteroidota, etc. (Figure 5C). Similarly, the community structure of wine grape samples was similar at the genus level (Figure 5D).

### 3.6 Comparative analysis of rhizosphere microbiota differences among different wine grape varieties

**Figure 6:**
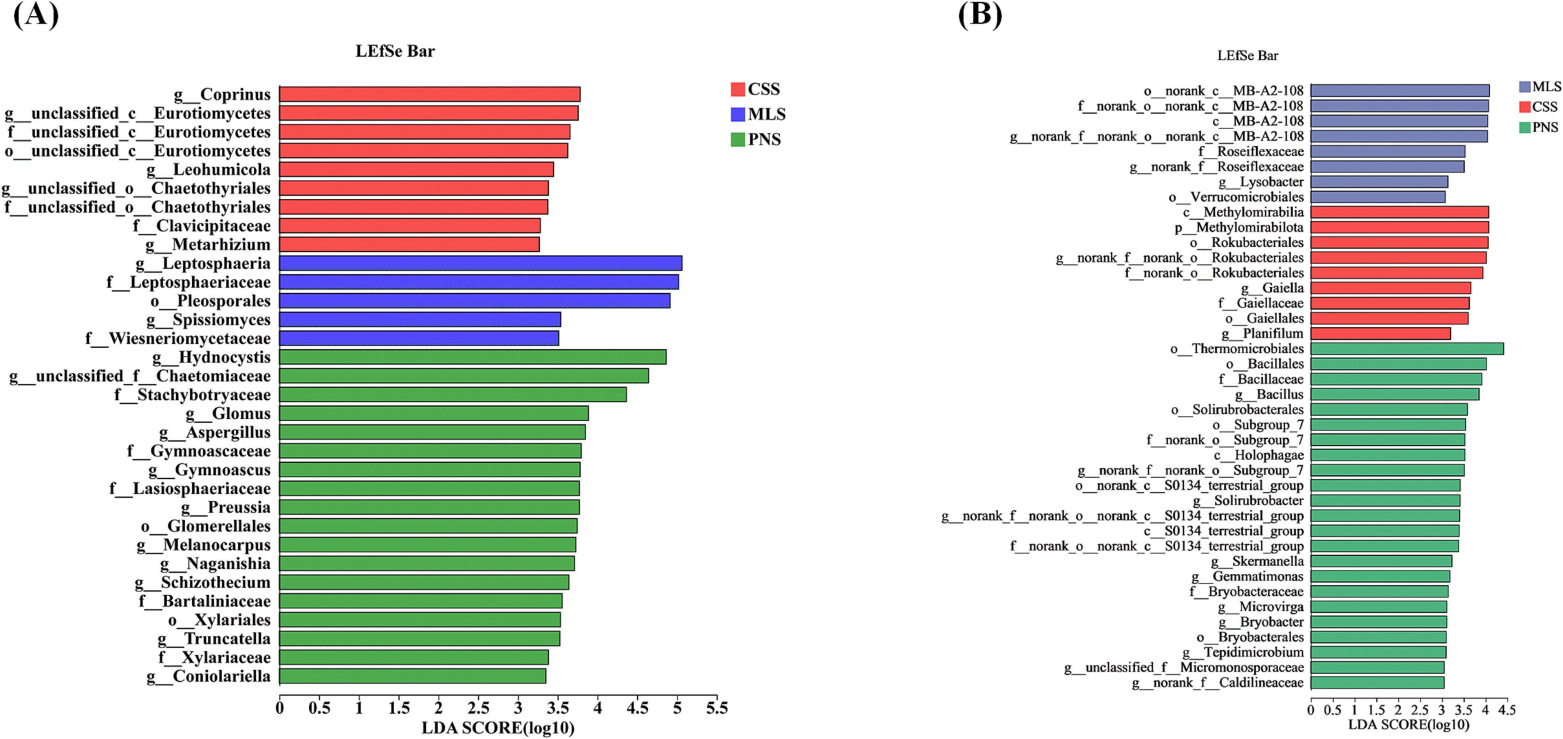
LEfSe analysis of rhizosphere soil microorganisms in wine grapes. **(A)** Fungal LEfSe. **(B)**: Bacterial LEfSe.

Differential microorganisms in the rhizosphere soil and roots of several grape types were screened using LEfSe (LDA > 3, *p* < 0.05) based on the phylum to genus level. In general, the rhizosphere soil of wine grapes contains more differential microorganisms than bacteria (Figure 6). d_Liposphaeria (LDA = 5.06, *p* = 0.03), f_Liposphaeriaceae (LDA = 5.02, *p* = 0.03), o_Pleosoporales (LDA = 4.91, *p* = 0.04), d_Hydnocystis (LDA = 4.87, *p* = 0.04), f_Chaetomiaceae (LDA = 4.64, *p* = 0.03), and d_Prinus (LDA = 3.78, *p* = 0.03) are the primary fungal differential microorganisms found in the rhizosphere soil of three wine grapes (Figure 6A). o_Thermomobiales (LDA = 4.41, *p* = 0.04), c_Methylmirabilia (LDA = 4.01, *p* = 0.03), and o’-Rokubacteres (LDA = 3.93, *p* = 0.03) are the primary differential microorganisms among rhizosphere soil bacteria (Figure 6B).

**Figure 7:**
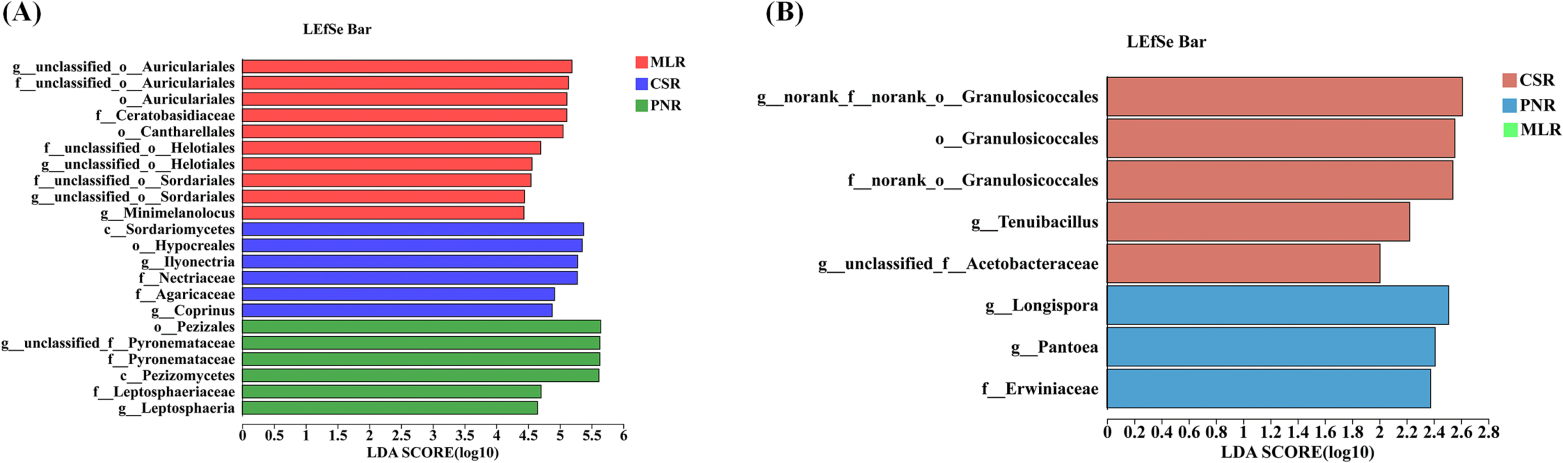
LEfSe analysis of endophytic microorganisms in wine grape roots. **(A)** Endophytic fungal LEfSe. **(B)** Endophytic Bacteria LEfSe.

Similarly, endophytic fungi in the roots of three different wine grape varietals were subjected to LefSe differential microbiological analysis. The results showed that the number of endophytic fungi with LefSe (LDA > 3, *p* < 0.05) was significantly more than that of endophytic bacteria, and the abundance of representative endophytic fungi had a significant impact on the differential effect (Figure 7). f_Pyronemataceae (LDA = 5.65, *p* = 0.02), o-Pezizomycetes (LDA = 5.66, *p* = 0.02), c_Pezizomycetes (LDA = 5.63, *p* = 0.02), and c_Sordariomycetes (LDA = 5.38, *p* = 0.03) are the primary microbiological differences in endophytic fungi (Figure 7A). Furthermore, the examination of LefSe (LDA > 2, *p* < 0.05) endophytic bacteria in wine grapes revealed a limited number of representative microorganisms exhibiting variations in endophytic bacteria, which were exclusively detected in Pinot Noir and Cabernet Sauvignon samples (Figure 7B).

### 3.7 Correlation analysis between quality indicators of wine grapes and rhizosphere microorganisms

**Figure 8:**
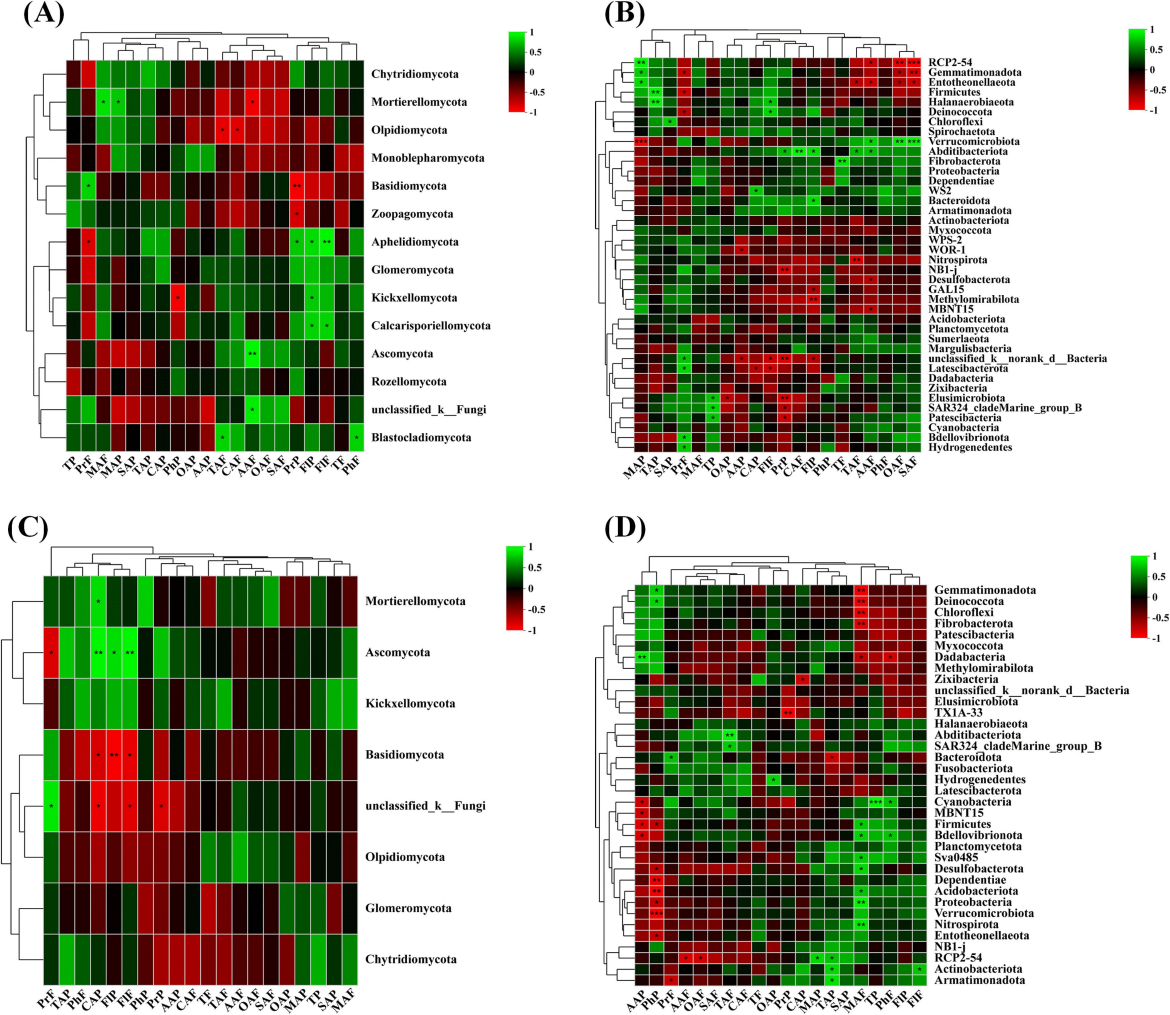
The correlation between organic acids and phenolic substances in the fruit flesh and peel of wine grapes and rhizosphere microorganisms at the Phylum level. **(A)** The heatmap of the correlation between fungal communities and organic acids and phenolic substances in the rhizosphere soil of wine grapes. **(B)** The heatmap of the correlation between bacterial communities and organic acids and phenolic substances in the rhizosphere soil of wine grapes. **(C)** The heatmap of the correlation between endophytic fungal communities in wine grape roots and organic acids and phenolic substances. **(D)** The heatmap of the correlation between endophytic bacterial communities in wine grape roots and organic acids and phenolic substances.

The correlation analysis between quality indicators and rhizosphere microbial diversity of wine grapes in the Hexi Corridor of China showed that at the phylum level, the Proanthocyanidin of wine grape fruit flesh was positively correlated with Basidiomycota (*R* = 0.70), but negatively correlated with Aphelidiomycota (*R* = −0.70). Malic acid in the fruit peel and flesh had a positive correlation with Mortierellomycota (*R* = 0.75, *R* = 0.67), while phenolic in the peel was negatively correlated with Kickxellomycota (*R* = −0.76). At the same time, it was found that tartaric acid and citric acid in the flesh were negatively correlated with Olpidiomycota (*R* = -0.72, *R* = −71). Conversely, Ascomycota and unclassified _ k__ Fungi positively correlated with ascorbic acid in the fruit flesh (*R* = 0.83, *R* = 0.78). Additionally, Aphelidiomycota was positively correlated with Proanthocyanidin in the peel and flavonol in both the fruit peel and flesh (*R* = 0.67, *R* = 0.74, *R* = 0.84). On the other hand, flavonol also showed a favorable correlation with Calcarisporiellomycota (*R* = 0.70, *R* = 0.71) (Figure 8A). PCP2-54, Gemmatimonadota, and Entotheonellaeota in the rhizospheric soil bacterial community were positively correlated with malic acid in the fruit peel (*R* = 0.88, *R* = 0.73, *R* = 0.78) and negatively correlated with oxalate acid (*R* = −0.87, *R* = −0.67, *R* = −0.66) and succinic acid (*R* = −0.97, *R* = −0.81, *R* = −0.77) in the fruit flesh. Verrucomicrobiota obtained extremely significant levels inits negative correlation with malic acid in fruit peel (*R* = 0.95) and its positive correlation with succinic acid and oxalate acid in fruit meat (*R* = 0.93, *R* = 0.85). In addition, Proanthocyanidin in the fruit peel had significant negative correlations with Elusimicrobiota, unclassified_k__norank_d__Bacteria, and NB1-j (*R* = −0.80, *R* = −0.81, *R* = −0.81) (Figure 8B). At the phylum level, in the endophytic fungal microbial community of wine grape roots, flavonol in the fruit peel and flesh and citric acid in the peel had a positive correlation with Ascomycota (*R* = 0.70, *R* = 0.83, *R* = 0.80), but negatively correlated with Basidiomycota (*R* = −0.72, *R* = −0.86, *R* = −0.86) (Figure 8C). In the endophytic bacterial community, ascorbic acid in the fruit peel was negatively correlated with various endophytic fungi such as Cyanobacteria (*R* = −0.71), MBNT15 (*R* = −0.72), Firmicutes (*R* = −0.77), and Bdellovibrionota (*R* = −0.75). Similarly, phenolics in the fruit peel were also negatively correlated with endophytic bacteria such as Desulfobacterota, Dependentiae, Acidobacteriota Verrucomicrobiota (*R* = −0.67, *R* = −0.82, *R* = −0.85, and *R* = 0.94). Differently, malic acid in the fruit flesh was positively correlated with various endophytic bacteria such as Nitrospirota and Proteobacteria (*R* = 0.80, *R* = 0.83), but negatively correlated with Gemmatimonadota, Deinococcota, Chloroflexi, and Fibrobacterota (*R* = −0.83, *R* = −0.85, *R* = −0.82, *R* = −0.80) (Figure 8D).

**Figure 9:**
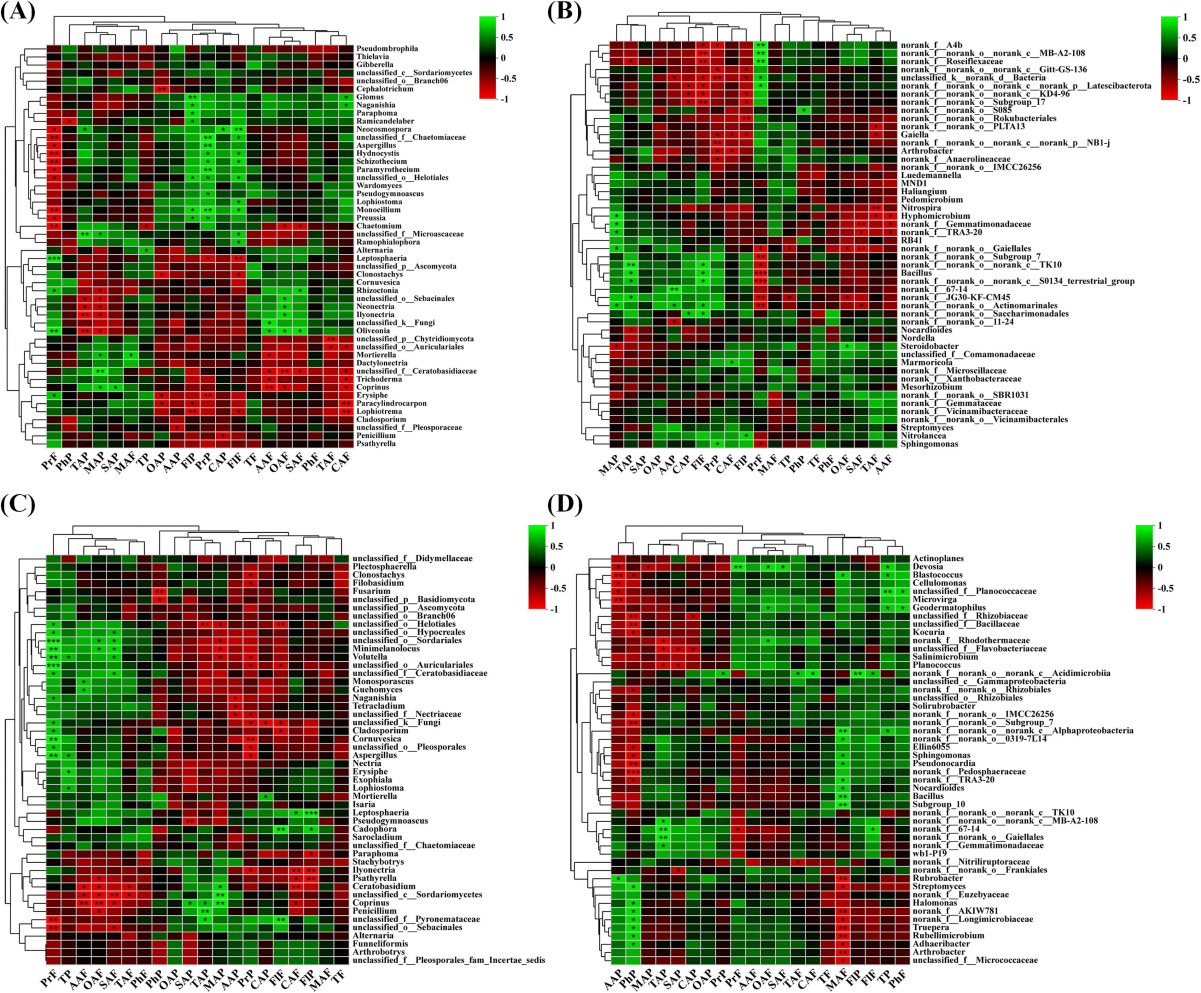
The correlation between organic acids and phenolic substances in the fruit flesh and peel of wine grapes and rhizosphere microorganisms at the Genus level. **(A)** The heatmap of the correlation between fungal communities and organic acids and phenolic substances in the rhizosphere soil of wine grapes. **(B)** The heatmap of the correlation between bacterial communities and organic acids and phenolic substances in the rhizosphere soil of wine grapes. **(C)** The heatmap of the correlation between endophytic fungal communities in wine grape roots and organic acids and phenolic substances. **(D)** The heatmap of the correlation between endophytic bacterial communities in wine grape roots and organic acids and phenolic substances.

At the genus level, in the rhizospheric soil fungal community of wine grapes, Proanthocyanidin in the fruit flesh was positively correlated with multiple fungal genera, such as unclassified _f__ Chaetomiaceae, *Hydnocystis* and *Schizothecium*, and positively correlated with *Leptosphaeria* and *Oliveonia* (*p* < 0.01). In addition, Proanthocyanidin in the peel and flavonol in the fruit peel and flesh were also positively correlated with multiple soil fungi. In contrast, ascorbic acid, oxalate acid, citric acid in the fruit flesh, and oxalate acid in the fruit peel were negatively correlated with multiple soil fungi (Figure 9A). In the rhizosphere soil bacterial community at the genus level, the overall negative correlation between wine grape quality and soil bacterial community was higher than the positive correlation (Figure 9B). At the genus level, Proanthocyanidin in the fruit flesh was positively correlated with multiple root endophytic fungal communities, such as *Hydnocystis* (*R* = −0.87) and *Schizothecium* (*R* = −0.81). In contrast, Proanthocyanidin in the peel was negatively correlated with root endophytic fungi. In addition, flavonol in the peel and citric acid in the fruit flesh were negatively correlated with *Paracylindrocarpon* and *Lophiotrema* (Figure 9C). At the genus level, ascorbic acid and phenolics in the fruit peel had a positive correlation with rhizospheric endophytic bacteria with high abundance, and the tannin in the fruit peel was positively correlated with endophytic bacteria with high abundances, such as *Devosia* and *Blastococcus*. In contrast, malic acid in the flesh had a negative correlation with endophytic bacteria *Rubrobacter*, *Streptomyces*, *Rubellimicrobium*, *Adhaeribacte*r, and *Arthrobacte* (Figure 9D).

## 4 Discussion

The wine grapes industry is one of the leading agricultural industries in the Hexi Corridor of China, playing an essential role in the local national economy (Fang et al., 2017). The manufacture of wine largely depends on the superior quality of grapes used for its making, and the variances between the grape kinds greatly influence the characteristics of different wine varieties (Steiner et al., 2021; Golan and Shalit, 1993). Plant rhizosphere microorganisms are a vital biological resource in regulating plant growth, metabolism, and other aspects (Paliwoda and Mikiciuk, 2020; Venturi and Keel, 2016; Shaw et al., 2006; Meena et al., 2017). However, there are limited reports on the relationship between rhizosphere microorganisms and the quality of different wine grape varieties.

Some reports have shown differences in rhizosphere microorganisms among various grape varieties (Pinto et al., 2014; del Carmen Portillo et al., 2016; Kecskeméti et al., 2016). Similarly, this study also observed significant differences in the rhizosphere microorganisms of three wine grape varieties. Among them, especially at the genus level, the fungal richness in the rhizosphere soil of Merlot was higher than that of Cabernet Sauvignon and Pinot Noir. In comparison, the bacterial richness in the rhizosphere soil of Cabernet Sauvignon was higher than that of Merlot and Pinot Noir. Lefse’s study also found different microorganisms in the rhizosphere of the three wine grape varieties. d_Liposphaeria and f__Leptosphaeriaceae were the main rhizospheric soil differential fungi, while o_Thermomomicrobiales and C__Methylomirabilia were differential bacteria. Similarly, f_Pyronemataceae, o__Pezizales were endophytic differential fungi. Because of genetic traits, root exudates, soil conditions, and other elements, the rhizosphere microbes of various wine grape types exhibit diversity (Zhang et al., 2019; Tao et al., 2024; Manici et al., 2017). Our study found that the distribution of microbial communities in the rhizosphere of the three wine grapes differed, which may be closely related to the genetic characteristics of grape varieties. This has a specific guiding significance for the actual production of wine grapes.

Organic acids are the primary source of wine acidity and constitute the skeleton and soul of wine. These organic acids include tartaric acid, malic acid, citric acid, succinic acid, and lactic acid. They exist in wine in a free state, giving the wine a fresh and refreshing feeling (Kliewer, 1970; Mato et al., 2005; Chidi et al., 2018; Amerine, 1964; Callejón et al., 2010). This study tested the quality indicators of different varieties of wine grapes and found significant differences in acid and phenolic substances among different wine grape varieties.

Oxalic acid (OA) is involved in the flavor composition of wine. Although its flavor is not prominent, it can work together with other organic acids, such as tartaric and malic acid, to create wine’s unique taste and flavor. In addition, OA also has specific antioxidant properties, which help protect wine from oxidative damage. During the aging process of wine, OA can work with other antioxidant substances to maintain the color and flavor stability of wine and extend its shelf life (Robles et al., 2019; Simson and Debolt, 2012; Wang et al., 2015). In this study, the OA content in the mature skins of three types of wine grapes was not significantly different, while a notable distinction was observed in the fruit flesh. Variations in the OA concentration of the fruit flesh may also reflect the changes in wine quality caused by the different grape varietals employed in winemaking. At the Phylum level, Verrucomicrobiota in wine grape rhizosphere soil was positively correlated with OA, while it was negatively correlated with soil and root endophytic strain pcp2-54. There are reports that grape samples from both cultivated and non-cultivated soils have a higher abundance of Verrucomicrobiota (Besze et al., 2024). Similarly, our study found verrucomicrobiota in the rhizosphere soil microbial composition of wine grapes at the phylum level.

Tartaric acid (TA) is one of the most abundant organic acids in wine, giving it a crisp taste (Yalcin et al., 2008). TA affects the acidity of the wine and interacts with other components to provide complex flavors and structures to the wine. It interacts with other acids and sugars in wine to create a balanced and harmonious taste, making it more layered and complex (Li et al., 2023; Zhao et al., 2023a, 2023b). During the aging process, TA can combine with tartrate salts to form crystals, which will deposit at the bottom of the bottle to prevent suspended solids from affecting the clarity of the wine. This stability helps maintain the quality and taste of wine, extending its shelf life (Cui et al., 2024; Edwards et al., 1985; Walker et al., 2004). In addition, the TA content can reflect the grapes’ maturity to a certain extent. With the ripening of grapes, the proportion of tartaric acid will gradually increase (Lamikanra et al., 1995; Saito and Kasai, 1968). The TA content of the fruit peel and flesh of three different wine grape varieties varied significantly in this investigation, with the fruit peel of Cabernet Sauvignon having the most outstanding TA amount. At the phylum level, the composition of Blastocladiomycota in the rhizosphere soil fungal community of wine grapes positively correlates with the fruit flesh’s TA content. In contrast, the bacterial communities Firmicutes and Halanaerobiaeota have a reasonable correlation with the TA content in the fruit peel. At the same time, the composition of Abditibacter in the endophytic bacterial community of wine grapes is positively correlated with the TA content in the fruit flesh. At the genus level, unclassifiedd_f_Microascaceae in the rhizosphere soil fungal community and norank_f_ norank_o norank_c TK10 in the bacterial community are positively correlated with TA in the fruit peel. Similarly, Penicillium and norank_f 67–14 and norank_f norank_o Gaiellale are also positively correlated with TA in the fruit peel (Figure 9). Similar to our research, the analysis of microbial diversity in soil and root samples of wine grapes showed that Proteobacteria and Actinobacteria were dominant in the rhizosphere of wine grapes and the effective promotion of wine grape quality by rhizosphere microorganisms (Rivas et al., 2022). This study found that multiple microbial communities in the rhizosphere of different varieties of wine grapes were positively correlated with TA, indicating that rhizosphere microorganisms may impact the quality of wine grapes and indirectly affect the production of wine.

Malic acid (MA) plays a crucial role in wine grapes, as it significantly affects the wine’s flavor, taste, and stability. MA has a complex and rich aroma, which can enhance the richness of the wine and improve its typicality and solidity. During the winemaking process, malic acid undergoes malic, lactic acid fermentation, which is converted into lactic acid and carbon dioxide, softening the initially sharp taste of malic acid and reducing the overall acidity of the wine, making it easier to drink (Kunkee, 1991; Su et al., 2014). For wines with aging potential, malolactic fermentation makes the body softer and the aroma more beautiful and delicate, enhancing the wine’s overall style (D’Onofrio et al., 2019). Our study observed significant differences in the MA content in the fruit peel of three types of wine grapes, while no significant differences were found in the fruit flesh. At the phylum level, Proteobacteria and Nitrospirota in the endophytic bacterial community of grape roots showed a positive correlation with MA in the pulp. In contrast, RCP2-54 in the rhizosphere soil bacterial community of wine grapes showed a positive correlation with MA in the pericarp. At the genus level, there was a positive correlation between MA in the fruit peel and unclassified_f_ceratobasidiaceae in the grape rhizosphere soil fungal community, as well as between unclassified_c_sordariomycetes and *Coprinus* in the grape endophytic fungal community. On the other hand, MA in the fruit flesh had a positive correlation with *bacillus* and subgroup_10 in the root endophytic bacterial population. Studies have shown that malic acid is vital in enriching grape nutrition (Kurt et al., 2018; Satisha and Somkuwar, 2019). This study found that there was a correlation between MA in wine grape pericarp and rhizosphere microbial community, and there were significant differences in MA in different wine grape fruit peel, indicating that MA in wine grape pericarp may play a role in reducing acidity in later wine brewing.

Ascorbic acid (AA) has strong reducibility and can be used as an antioxidant and oxygen scavenger to prevent or reduce the damage to wine flavor and pigment caused by oxidation (Oyuela Aguilar et al., 2020). In wine fermentation, ascorbic acid can promote yeast activity, maintain the appropriate redox potential, and improve fermentation efficiency. In addition, ascorbic acid is usually used together with SO_2_ to enhance the antioxidant effect of wine (Bradshaw et al., 2011; Skouroumounis et al., 2005). At the same time, an appropriate amount of AA can also improve the taste and flavor of wine (Peng et al., 1998; Barril et al., 2016). Our study found significant differences in AA content in the fruit peel and flesh of the three wine grapes, indicating that AA may play a role in regulating the quality of wine grapes. The correlation analysis between AA content and rhizosphere microorganisms showed that Ascomycota in grape rhizosphere soil fungal community was positively correlated with AA in fruit flesh of verrucomicrobiota and abditibacteriota in the bacterial community at the phylum level. At the same time, the endophytic bacteria dadabacter in grape root showed a favorable correlation with AA in the pericarp. At the genus level, unclassified_k_fungi and *Oliveonia* in grape rhizosphere soil had a positive correlation AA. The endophytic fungal communities in grape roots such as Monosporascus and Guehomyces were also positively correlated with AA in fruit flesh. Some studies have shown that ascorbic acid impacts the color and aroma of wine, and the moderate presence of ascorbic acid is conducive to improving the quality of wine (Skouroumounis et al., 2005; Landbo and Meyer, 2001). In addition, AA also positively affected the agronomic and physiological indexes of grape growth (Khan et al., 2020; William et al., 1979). In our study, the correlation analysis between microbial diversity and AA showed that the AA content in both fruit flesh and peel was correlated with the rhizosphere microbial community of wine grapes, indicating that rhizosphere microbial activity plays a role in regulating grape biochemical characteristics, thereby affecting grape quality and indirectly affecting wine quality.

Citric acid (CA) plays an important role in regulating the acidity of wine, preventing oxidation, and removing excess metal ions such as iron and copper from wine (Minjares-Fuentes et al., 2014; Soyer et al., 2003). Furthermore, CA also plays a vital role in regulating soil pH and enhancing grape disease resistance (Huang et al., 2021). According to this study, the CA content in the fruit flesh of three different wine grape varieties varied significantly, with Merlot having the most incredible content. At the phylum level, a positive correlation existed between the Abditibacteria of the grape rhizosphere soil bacterial community and the CA in the fruit flesh. In contrast, the Ascomycota of the grape root endophytic fungal community positively correlated with the CA in the fruit peel. At the genus level, the correlation between CA content in fruit flesh and peel and Rhizosphere Soil and endophytic microbial community was weak, and rhizosphere microorganisms had little effect on grape CA.

Some studies have shown that succinic acid (SA) in wine grapes enriches the taste experience of wine and enhances its flavor stability and mellowness. Therefore, SA is one of the indispensable and important ingredients in the winemaking process (Thoukis et al., 1965; Lamikanra, 1997). Our study found significant differences in the SA content in the fruit flesh and peel of the three wine grapes. The correlation analysis between SA and grape rhizosphere microbial diversity showed that at the phylum level, Verrucomicrobiota in the rhizosphere soil bacterial community had a favorable correlation with SA in the fruit flesh. Conversely, the correlation between the endophytic bacterial community and SA was insignificant. The distinction was that SA in the fruit flesh was associated with several fungal genera in the grape rhizosphere microbial population at the genus level. Some studies have shown that multiple microorganisms can ferment to produce SA, indicating that microorganisms play a critical role in SA production (Song and Lee, 2006; Beauprez et al., 2010). Rhizosphere microorganisms are an important type of plant growth-promoting bacteria that regulate SA in wine grapefruits. So more research is necessary in this area in the future.

Tannins (T) build a stable, solid, and full-bodied “skeleton” for wine, giving it a sense of structure, weight, and texture, bringing rich taste to wine and determining its flavor (McRae and Kennedy, 2011). Meanwhile, tannins have antioxidant properties, allowing red wines rich in tannins to mature over the years (Neves et al., 2010). During the fermentation and aging process of wine, the higher the T content, the darker the color (Ma et al., 2014). At the same time, an appropriate amount of tannins can improve the quality of wine, making its taste fuller and more layered (Herderich and Smith, 2005). However, the T level of the three grape varietals used to make wine did not differ significantly between the peel and flesh, according to this study. The T concentration in grape peel and flesh and the fungus populations in grape rhizosphere soil and roots do not differ significantly at the phylum level. Nonetheless, there is a strong correlation between the rhizosphere’s bacterial communities, including Elusimicrobiota, SAR324_cladeMarine_group_B, Patescibacteria, Cyanobacteria, and tannins found in fruit peels. At the genus level, some communities of rhizosphere microorganisms of the three wine grapes, such as Alternaria, Nectria, Erysiphe, Lophiostoma, and Devosia, were positively correlated with tannins in fruit peel but did not correlate with the fruit flesh. According to reports, tannins have an impact on grape maturity in addition to wine quality (Bindon et al., 2014; Kyraleou et al., 2017). Nonetheless, this study found no discernible variation in the amount of T in the peel and flesh of the various wine grape varieties, suggesting that the variety differences had little effect on T accumulation and that there may be a relationship between rhizosphere microorganisms and wine grape T content.

Flavonols (Fl) are important phenolic compounds in wine, which can interact with anthocyanins to form stable complexes, enhancing the color of wine and improving its stability during the brewing process (Mazza et al., 1999; Li et al., 2020). As a co-pigment, Fl play a complementary role in fresh red wine, and their content and type significantly impact the wine’s overall quality (Makris et al., 2006). Furthermore, Fl have antioxidant activity and can protect grape fruits from UV damage (Kolb et al., 2003). According to our research, there were notable variations in the amount of Fl in the fruit peel and flesh of various grape varieties during the mature stage of winemaking. In particular, the peel of Pinot Noir had the highest flavonoid content, while Merlot had the lowest. At the phylum level, multiple bacterial and fungal communities in the rhizosphere soil and roots of three different wine grapes showed significant correlations with Fl, with the most positively correlated. Surprisingly, similar results were also observed at the genus level. Scholars have compared phenolic compounds in different grape varieties and found differences in Fl, possibly due to differences between grape varieties (Fanzone et al., 2012; Liang et al., 2012). The phenolic chemicals found in wine produced from various grape varieties vary, maybe due to genetic variations among grape varieties (Silva et al., 2023). The variations in Fl between the various wine grape varieties in this study may affect subsequent wine fermentation, and the association between Fl and grape rhizosphere microorganisms also implies that rhizosphere microorganisms may be a significant factor influencing flavonols.

Proanthocyanidin (Pr) is an important pigment in red wine, giving it its ruddy color. Pr in grape peels will dissolve into grape juice, and after fermentation and aging, they form the unique color of wine (Cerpa-Calderón and Kennedy, 2008). Pr can also increase the astringency and structure of wine, making the taste fuller and more layered. At the same time, Pr can also combine with other ingredients in wine to form stable compounds, thereby extending the shelf life of wine (Garrido-Bañuelos et al., 2022). In this study, the Pr content in the fruit flesh and peel of the three wine grapes showed significant differences, and Pr in the peel was much higher than that in the flesh, which confirmed that Pr in the peel was essential for wine grapes. A range of rhizosphere microorganisms in grapes was linked to Pr content at the phylum and genus levels, suggesting that rhizosphere microorganisms may be crucial in controlling grape Pr and that their diversity influences wine grape quality.

The content and type of total phenolic (Ph) also significantly impact the quality of wine, as they can affect the wine’s color, taste, and stability. For example, Ph can interact with anthocyanins in wine to form stable complexes, enhancing the color of the wine (Waterhouse, 2002; Yue et al., 2021). Studies have shown a positive correlation between total phenols and glucose content in grapes during the ripening stage (Pirie and Mullins, 1977). Our research found a significant difference in the Ph content in the flesh of wine grapes, while the peel had a greater Ph level than the flesh, but the difference was not statistically significant. Specific bacterial phyla in the endophytic bacterial community of grape roots strongly correlated with Ph content at the phylum level, and the same was true for grape rhizosphere microorganisms and Ph at the genus level. The research results indicate that the diversity of rhizosphere bacterial communities may affect the accumulation of Ph in wine grapes.

## 5 Conclusion

The plant rhizosphere is a complex system, and the rhizosphere microbial community plays an important role in regulating plant production and development. The quality characteristics of wine grapes have a key impact on wine brewing. This study used high-throughput sequencing to analyze the rhizosphere soil and endophytic fungal and bacterial communities of three wine grape varieties: Merlot, Cabernet Sauvignon, and Pinot Noir. The rhizosphere microbial community structure of different wine grape varieties was found to be different. The contents of several organic acids and phenols in the fruit peel and flesh of three wine grape varieties at maturity were detected. It was discovered that there were significant differences in the contents of organic acids and phenols among different grape varieties. The correlation analysis between the contents of grape organic acids, phenols, and rhizosphere microorganisms showed a significant correlation between the indexes and some rhizosphere microorganisms. The related research results provided an important theoretical basis for improving the quality of wine grape and wine brewing. Meanwhile, one of the most compelling scientific challenges ahead is unraveling the intricate ways in which rhizosphere microorganisms influence the quality of wine grapes.

## Data Availability

The datasets presented in this study can be found in online repositories. The names of the repository/repositories and accession number(s) can be found at: https://www.ncbi.nlm.nih.gov/, PRJNA1197982.

## Footnotes

^1^ https://cloud.majorbio.com
