# Supplementary material for: Correlation analysis between quality characteristics and rhizosphere microorganisms of different wine grape varieties during their ripening phase
Source: Front Microbiol. 2025 Mar 26;16:1546323. doi: 10.3389/fmicb.2025.1546323 (PMC11979242; doi:10.3389/fmicb.2025.1546323)
Supplement: Supplementary file 1 [file Table_1.docx]

**TABLE S1. Gradient standard solution for six organic acids**

| **Organic Acid (g/L)** | **Concentration Gradient** | | |
| --- | --- | --- | --- |
|  | **1** | **2** | **3** |
| Oxalic Acid | 0.0502 | 0.1004 | 0.2088 |
| Tartaric Acid | 0.1538 | 0.3076 | 0.6125 |
| Malic Acid | 0.3506 | 0.7012 | 1.4024 |
| Ascorbic Acid | 0.0518 | 0.1036 | 0.2072 |
| Citric Acid | 0.184 | 0.920 | 1.840 |
| Succinic Acid | 0.2215 | 0.443 | 0.886 |

**TABLE S2 The representative sequence information of OTUs**

| **Sequence Type** | **Sample** | **Seq_num** | | **Base_num** | **Mean_length** | **Min_length** | **Max_length** | **OTU_num** | **Sequences** |
| --- | --- | --- | --- | --- | --- | --- | --- | --- | --- |
| ITS sequence of rhizosphere soil | HS1 | 145531 | | 35549757 | 244.276182 | 153 | 538 | 213 | 51751 |
|  | HS2 | | 137982 | 34624249 | 250.933085 | 128 | 538 | 235 | 51751 |
|  | HS3 | 110833 | | 28056801 | 253.144831 | 171 | 523 | 185 | 51751 |
|  | CS1 | 177304 | | 45012875 | 253.873996 | 88 | 519 | 244 | 51751 |
|  | CS2 | 129690 | | 32606809 | 251.42115 | 163 | 519 | 201 | 51751 |
|  | CS3 | 128779 | | 31753700 | 246.57514 | 148 | 506 | 185 | 51751 |
|  | MS1 | 177284 | | 41858523 | 236.109987 | 133 | 534 | 242 | 51751 |
|  | MS2 | 179925 | | 38845187 | 215.896551 | 53 | 537 | 185 | 51751 |
|  | MS3 | 144821 | | 35298723 | 243.740362 | 116 | 517 | 189 | 51751 |
| 16S rRNA sequence of rhizosphere soil | HS1 | 84331 | | 35154817 | 416.867071 | 247 | 534 | 3302 | 49108 |
|  | HS2 | 78416 | | 32719140 | 417.250816 | 221 | 452 | 3227 | 49108 |
|  | HS3 | | 64287 | 26886847 | 418.231478 | 310 | 469 | 3034 | 49108 |
|  | CS1 | 82432 | | 34494412 | 418.458997 | 219 | 525 | 3230 | 49108 |
|  | CS2 | 55558 | | 23236176 | 418.232766 | 262 | 452 | 2980 | 49108 |
|  | CS3 | 73023 | | 30575563 | 418.711406 | 317 | 524 | 3063 | 49108 |
|  | MS1 | 69451 | | 28963619 | 417.036745 | 250 | 469 | 3141 | 49108 |
|  | MS2 | 85027 | | 35580545 | 418.461724 | 218 | 519 | 3075 | 49108 |
|  | MS3 | 71246 | | 29756613 | 417.660121 | 284 | 477 | 3329 | 49108 |
| ITS sequence of grape roots | HR1 | 100697 | | 25931188 | 257.5169866 | 50 | 367 | 82 | 74543 |
|  | HR2 | 89984 | | 23743809 | 263.8670097 | 148 | 404 | 67 | 74543 |
|  | HR3 | 74566 | | 19632496 | 263.2901859 | 211 | 398 | 36 | 74543 |
|  | CR1 | 80986 | | 21008639 | 259.41075 | 170 | 467 | 79 | 74543 |
|  | CR2 | 106488 | | 24407688 | 229.205995 | 50 | 393 | 591 | 74543 |
|  | CR3 | 76110 | | 20212369 | 265.5678492 | 171 | 500 | 30 | 74543 |
|  | MR1 | 89465 | | 21457472 | 239.8420835 | 84 | 367 | 51 | 74543 |
|  | MR2 | 80088 | | 19059845 | 237.9862776 | 50 | 382 | 588 | 74543 |
|  | MR3 | 77282 | | 18435028 | 238.5423255 | 50 | 367 | 542 | 74543 |
| ITS sequence of rhizosphere soil | HR1 | 92638 | | 34941605 | 377.184363 | 352 | 461 | 3362 | 80780 |
|  | HR2 | 88060 | | 33219846 | 377.24104 | 356 | 442 | 3290 | 80780 |
|  | HR3 | 93957 | | 35426128 | 377.046181 | 356 | 442 | 3555 | 80780 |
|  | CR1 | 97246 | | 36670873 | 377.093896 | 354 | 442 | 3486 | 80780 |
|  | CR2 | 102853 | | 38798274 | 377.220635 | 319 | 442 | 3437 | 80780 |
|  | CR3 | 94426 | | 35578233 | 376.784286 | 319 | 466 | 3392 | 80780 |
|  | MR1 | 103614 | | 39063072 | 377.005733 | 353 | 441 | 3464 | 80780 |
|  | MR2 | 111900 | | 42205964 | 377.175728 | 353 | 442 | 3383 | 80780 |
|  | MR3 | 100444 | | 37875528 | 377.08104 | 325 | 442 | 3390 | 80780 |

**TABLE S3 The taxonomic numbers of fungi and bacteria in the samples**

| Type | Fungi | | | | |  | Bacteria | | | | |
| --- | --- | --- | --- | --- | --- | --- | --- | --- | --- | --- | --- |
|  | Phylum | Class | Order | Family | Genus |  | Phylum | Class | Order | Family | Genus |
| Soil | 14 | 34 | 71 | 142 | 148 |  | 40 | 135 | 330 | 511 | 925 |
| Root | 8 | 19 | 41 | 73 | 110 |  | 36 | 109 | 261 | 467 | 952 |


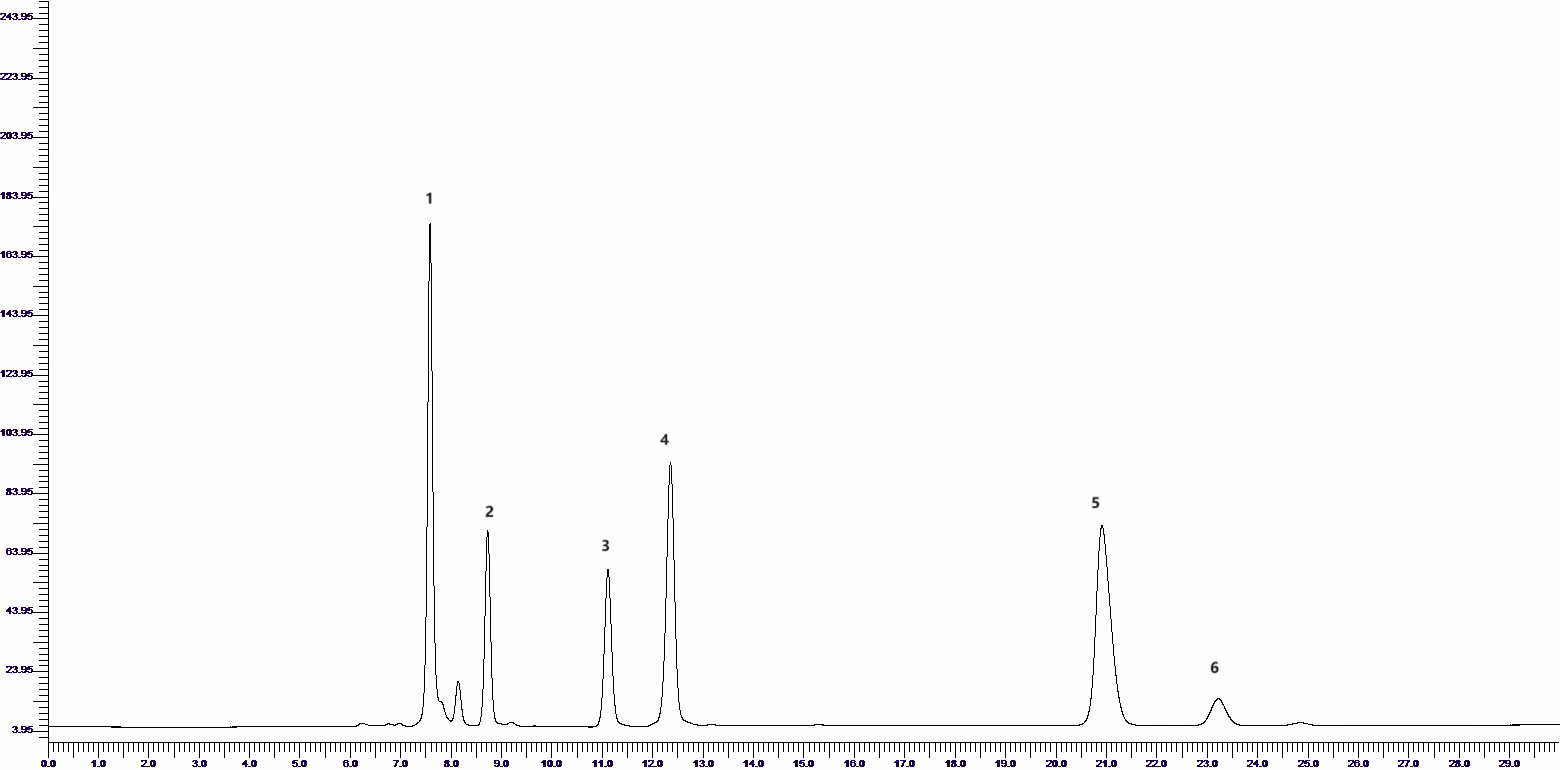


**FIGURE S1 The chromatogram of the organic acid mixed standard solution**
